# An unusual cause of meningismus

**DOI:** 10.11604/pamj.2013.16.28.3350

**Published:** 2013-09-26

**Authors:** Zouheir Hafidi, Rajae Daoudi

**Affiliations:** 1 Université Mohammed V Souissi, Service d'Ophtalmologie A de l'hôpital des spécialités, Centre hospitalier universitaire, Rabat, Maroc

**Keywords:** Vogt-Koyanagi-Harada disease, autoimmune disorder

## Image in medicine

Vogt-Koyanagi-Harada disease is an uncommon autoimmune disorder. Its pathophysiology is related to T-cell-mediated autoimmune processes directed against one or more antigenic components of melanocytes. The diagnosis of this condition is primarily based on clinical features including: ocular findings (granulomatous uveitis, choroiditis, exsudative retinal detachment, papillitis) neurological (meningismus, tinnitus, cerebrospinal fluid pleocytosis) and integumentary manifestations (alopecia, poliosis, vitiligo). We report the case of a 25 years old woman presented with 1 month history of painful vision loss with tinnitus and headache. She didn't report any previous ocular trauma or surgery. At examination there was a bilateral mild anterior chamber inflammation. Dilated funduscopy (Panel A) revealed mild vitritis with multiple yellowish deep retinal lesions at the level of the retinal pigment epithelium (white arrowheads) and optic disc hyperemia (arrows). At general examination we noted a temperature of 38 ° with a painful stiff neck. There were also some areas of cutaneous depigmentation (Panel B) on the forehead and nose bridge. Cranial computed tomography was unremarkable. A lumbar puncture revealed a sterile liquid with pleocytosis (25 mononuclear cells/dL). A work-up was performed including: ANCA C and P, Compete -Blood- Count, erythrocyte sedimentation rate, Rheumatoid factor, angiotensin conversing enzyme, lysozyme, Lyme titers, Interferon-gamma release assays, Rapid Plasma Reagin, all of which were unrevealing. Fluorescein angiography (Panel C) revealed multiple punctate hyperfluorescent dots (white arrowheads) with dye leakage and disc hyperfluorescence indicating severe papillitis (black arrowheads). Findings on audiometry were within normal limits. The patient was diagnosed with VKH disease. High dose methylprednisolone pulse was started (10mg/kg/day), followed by 60 mg prednisolone and 75 mg of cyclosporine per day with progressive tapering of oral steroids. Visual acuity improved to 20/25 after 1month with complete resolution of the intraocular inflammation.

**Figure 1:**
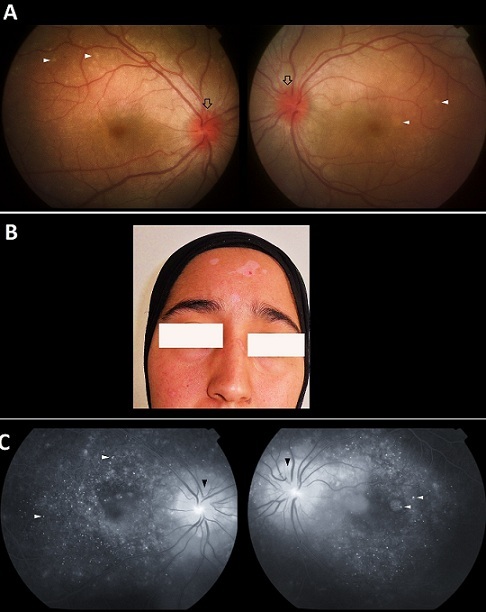
(A): Funduscopy showing a bilateral optic disc hyperhemia (arrows) with multiple yellowish lesions at the level of the retinal pigment epithelium (white arrowheads); (B): multiple depigmentation patches on the forehead and the bridge of the nose; (C): fluorescein angiography revealing multiple pinpoint areas of hyperfluorescence with a “starry sky” appearance

